# The combination therapy of oncolytic virotherapy

**DOI:** 10.3389/fphar.2024.1380313

**Published:** 2024-04-25

**Authors:** Yue Wang, Mengying Zhu, Huanyu Chi, Yang Liu, Guilin Yu

**Affiliations:** ^1^ Department of General Surgery, Cancer Hospital of China Medical University, Liaoning Cancer Hospital & Institute, Shenyang, China; ^2^ Department of Clinical Integration of Traditional Chinese and Western Medicine, Liaoning University of Traditional Chinese Medicine, Shenyang, China; ^3^ The Second Clinical College of Dalian Medical University, Dalian, China; ^4^ Department of Ophthalmology, First Hospital of China Medical University, Shenyang, China

**Keywords:** oncolytic viruses, combined therapy, tumor, cytokine, chemotherapy

## Abstract

**Introduction:** Compared to other cancer immunotherapies, oncolytic viruses possess several advantages, including high killing efficiency, excellent targeting capabilities, minimal adverse reactions, and multiple pathways for tumor destruction. However, the efficacy of oncolytic viruses as a monotherapy often falls short of expectations. Consequently, combining oncolytic viruses with traditional treatments to achieve synergistic effects has emerged as a promising direction for the development of oncolytic virus therapies.

**Methods:** This article provides a comprehensive review of the current progress in preclinical and clinical trials exploring the combination therapies involving oncolytic viruses.

**Results:** Specifically, we discuss the combination of oncolytic viruses with immune checkpoint inhibitors, chemotherapy, targeted therapy, and cellular therapy.

**Discussion:** The aim of this review is to offer valuable insights and references for the further advancement of these combination strategies in clinical applications. Further research is necessary to refine the design of combination therapies and explore novel strategies to maximize the therapeutic benefits offered by oncolytic viruses.

## 1 Introduction

Malignant tumors represent a global public health concern and are the second leading cause of human death worldwide (Siegel et al., 2023). Although there has been a downward trend in the incidence and mortality rates of malignant tumors, the mortality rate of certain malignancies, such as pancreatic cancer, liver cancer, and lung cancer, remains high. Currently, first-line treatments for malignant tumors include surgery, chemotherapy, radiation therapy, targeted therapy, and immunotherapy, but they are subject to various limitations. Surgery remains the only curative treatment for malignant tumors, but most patients are diagnosed at an advanced stage and have lost the opportunity for curative resection (Miller et al., 2022). Moreover, surgical tolerance and tumor characteristics, such as location, invasiveness, and microvascular metastasis, can significantly affect the implementation and efficacy of surgery. Traditional radiation therapy and chemotherapy cause significant damage to normal tissues in addition to targeting the tumor due to their low tumor specificity (Jhawar et al., 2023). Immunotherapy stimulates the body’s anti-tumor immunity, improves the tumor immune microenvironment, and exerts anti-tumor effects (Reck et al., 2022). However, due to the diverse immunogenicity of tumors, immunotherapy currently demonstrates efficacy against only a limited number of tumor types and can give rise to a range of immune-related adverse reactions. One significant concern is the occurrence of cytokine storms, which are characterized by an excessive release of pro-inflammatory cytokines. Cytokine storms can result in severe systemic inflammation and lead to organ damage or even failure (Bhardwaj et al., 2022).

As an emerging immunotherapy for tumors, oncolytic viruses selectively replicate within tumor cells to exert their anti-tumor effects while ensuring the safety of normal host cells (Enow et al., 2023; Lin et al., 2023). As an innovative modality of immunotherapy in the field of tumor treatment, oncolytic viruses have garnered increasing attention. These unique viruses possess the remarkable ability to selectively replicate within tumor cells, thus exerting potent anti-tumor effects. What sets oncolytic viruses apart is their capacity to specifically target cancerous cells while leaving normal host cells unharmed, ensuring the safety and wellbeing of the patient. This targeted replication within tumor cells not only leads to their destruction but also triggers an immune response, further enhancing the body’s natural defenses against cancer. This dual mechanism of action holds great promise for the development of effective and safe treatments for various types of tumors.

Two primary mechanisms underlie the functionality of oncolytic viruses. Firstly, they engage in robust replication within tumor cells, eventually resulting in tumor cell death via lysis. Secondly, they induce tumor cell lysis, thereby releasing damage-associated molecular patterns, tumor-associated antigens, and pathogen-associated molecular patterns. This activation subsequently triggers systemic anti-tumor immune responses. Several viruses, including herpes simplex virus, adenovirus, vaccinia virus, and measles virus, have undergone extensive study and genetic engineering to acquire selective oncolytic capabilities. Another category comprises wild-type and naturally attenuated viral strains, such as Newcastle disease virus and reovirus (Yao et al., 2023). Currently, monotherapy with oncolytic viruses has shown promising results in various cancers (Chen et al., 2023; Lovatt and Parker, 2023). However, in some phase II clinical trials with larger sample sizes, monotherapy with oncolytic viruses has not demonstrated satisfactory performance (Barton et al., 2021; Hajda et al., 2021; Garcia-Carbonero et al., 2022). In terms of safety, the main adverse reactions associated with oncolytic viruses include fever, chills, gastrointestinal reactions, flu-like symptoms, and overall safety is better than other immunotherapy drugs. Regarding efficacy, monotherapy with oncolytic viruses has shown certain therapeutic effects in some cancers with good immunogenicity, but overall performance falls short of expectations. The new generation of oncolytic virus formulations carrying multiple immune-stimulating exogenous factors are mostly still in clinical trial stages (Kazemi Shariat Panahi et al., 2022; Wang et al., 2024). Therefore, one of the development directions for current oncolytic virus therapies is to achieve synergistic efficacy by combining them with traditional treatment methods.

## 2 The combination of oncolytic virotherapy and immune checkpoint inhibitor

### 2.1 Combination of oncolytic viruses with PD-1/PD-L1 inhibitors

In the realm of combination therapy, the integration of oncolytic viruses with immune checkpoint inhibitors (ICIs) has emerged as a highly prominent and promising strategy. By combining these two potent therapeutic approaches, researchers aim to harness their complementary mechanisms of action to achieve enhanced anti-tumor effects. PD-1 and PD-L1 inhibitors are the most widely used ICIs and have shown promising results in the treatment of various solid tumors. However, their efficacy in tumors with complex immune suppressive microenvironments, such as pancreatic cancer, glioblastoma, and intrahepatic cholangiocarcinoma, remains unsatisfactory. Theoretically, the combination of oncolytic viruses and PD-1/PD-L1 inhibitors can overcome these limitations by improving the tumor immune microenvironment. In preclinical studies, the novel oncolytic virus VG161, when used in combination with PD-1 monoclonal antibody, demonstrates significant synergistic effects in a mouse model of pancreatic cancer (Deng et al., 2023; Shen et al., 2023). Single-cell sequencing and flow cytometry experiments have revealed that VG161 promotes the infiltration of CD8^+^ T cells and natural killer (NK) cells into the tumor, thereby altering the tumor’s immune microenvironment and providing a conducive setting for the subsequent use of PD-1 monoclonal antibody. Building upon these results, the phase I/II clinical trials vestigated the combination of VG161 and nivolumab in the treatment of advanced pancreatic cancer. Preliminary results have shown good safety and certain efficacy, with an extended median survival time compared to historical control groups. Additionally, a separate preclinical study has demonstrated that the novel oncolytic virus CF33, in combination with PD-L1 monoclonal antibody, elicits sustained anti-tumor immune responses and prolongs the survival of mice in a colon cancer model (Kim et al., 2021).

### 2.2 Expanding the use of other immune checkpoint inhibitors (ICIs)

Apart from PD-1/PD-L1 monoclonal antibodies, other immune checkpoint inhibitors (ICIs), such as cytotoxic T lymphocyte antigen 4 (CTLA-4), T cell immunoreceptor with immunoglobulin and ITIM domains (TIGIT), immune checkpoint T cell immunoglobulin and mucin domain 3 (TIM-3), and lymphocyte activation gene 3 (LAG-3), are reported to be upregulated in tumor-infiltrating lymphocytes. Consequently, this upregulation induces the suppression of the immune microenvironment and exacerbates the exhaustion of CD8^+^ T cells (Binnewies et al., 2018). In a preclinical study (Sugawara et al., 2021), the researchers discovered that the coadministration of intratumoral G47Δ and systemic anti-CTLA-4 antibody effectively mobilized effector T cells into the tumor, concurrently reducing regulatory T cells. Moreover, the combination therapy elicited significant upregulation of diverse gene signatures associated with inflammation, lymphoid lineage, and T cell activation. This observation suggests a potential transformation of immune-insusceptible tumors into an immune-susceptible state. Ultimately, this treatment improved the tumor immune microenvironment. Furthermore, zuo, et al. (Zuo et al., 2021) reported that VV-scFv-TIGIT acted synergistically with PD-1 or LAG-3 blockade, culminating in complete tumor regression in cases where tumors exhibited limited response to either VV treatment alone or immune checkpoint blockade monotherapy.

In a phase I clinical trial conducted in 2017, the combination therapy of T-VEC and pembrolizumab exhibited remarkable efficacy in patients with advanced melanoma, achieving a high objective response rate (ORR) of 62% and a complete response rate (CR) of 33% (Ribas et al., 2017). These findings underscored the potential application of oncolytic virotherapy in enhancing the effectiveness of anti-PD-1 therapy through modulation of the tumor microenvironment. However, the KEYNOTE-034 study revealed that the combination of T-VEC and pembrolizumab did not significantly improve progression-free survival (PFS) or overall survival (OS) compared to the control group (Chesney et al., 2023). Conversely, results from another phase I clinical trial indicated that the combination of T-VEC and ipilimumab demonstrated tolerable safety and appeared to exhibit superior efficacy compared to T-VEC or ipilimumab monotherapy (Puzanov et al., 2016). Additionally, a phase II clinical trial investigating the combination of T-VEC and pembrolizumab in advanced sarcoma patients also demonstrated encouraging outcomes, with an overall ORR of 35% (Kelly et al., 2020). Currently, numerous global clinical trials are exploring various combinations of oncolytic viruses with immunotherapies, with many encouraging results being presented at conferences. Among these strategies, the combination of oncolytic viruses with immunotherapies holds significant promise.

## 3 The combination of oncolytic virotherapy and chemotherapy

### 3.1 Challenges of combining oncolytic viruses with chemotherapy

The earliest combination therapy involving oncolytic viruses was with chemotherapy, but this strategy has been met with mixed success and remains a topic of debate. While chemotherapy is a well-established treatment for many types of cancer, its use in combination with oncolytic viruses has not proven to be consistently effective. In theory, chemotherapy can improve the tumor’s immune microenvironment, and subsequent use of immunotherapy after chemotherapy may achieve synergistic effects (Wu et al., 2019). However, the replication of oncolytic viruses mainly depends on active tumor cells. If chemotherapy is administered first before using oncolytic viruses, it would be difficult for oncolytic viruses to obtain an ideal survival environment when most tumor cells are killed by chemotherapy drugs, as the basis for the action of oncolytic viruses is their effective replication within tumor cells. Conversely, there is also a certain controversy over administering oncolytic viruses before chemotherapy, as the anti-tumor immune cells activated by oncolytic viruses may be killed by chemotherapy drugs (Driscoll et al., 2020).

### 3.2 Clinical challenges and efficacy in combining oncolytic viruses with chemotherapy

Despite some promising results observed in combining oncolytic viruses with chemotherapy (Mahalingam et al., 2020; Moreno et al., 2021), several clinical trials with large sample sizes have demonstrated poor efficacy for this strategy. For instance, a study (Eigl et al., 2018) revealed that the combination of pelareorep and docetaxel exhibited tolerable adverse events and similar disease progression but inferior response rates and overall survival, thus not justifying further investigation. In advanced prostate cancer patients, the addition of oncolytic virus to docetaxel resulted in a median survival time of 19.1 months, whereas docetaxel monotherapy yielded a median survival time of 21.1 months. Another study (Jonker et al., 2018) showcased the tolerability of combining pelareorep with FOLFOX/BEV, leading to an increased objective response rate (ORR), but inferior progression-free survival (PFS). Subgroup analysis based on baseline variables, including the Kirsten rat sarcoma oncogene, failed to identify any groups benefiting from PFS. The lack of benefit with pelareorep may be attributed to reduced treatment intensity with conventional agents. Besides, Arnold, et al. (Arnold et al., 2022) reported that the combination of pela with chemotherapy and a checkpoint inhibitor demonstrated favorable tolerability. Meeting the primary efficacy endpoint, the notable high objective response rate (ORR) and clinical benefit rate (CBR) observed in this study present a promising outlook, surpassing tumor response benchmarks set by previous first-line PDAC treatment investigations.

## 4 The combination of oncolytic virotherapy and targeted therapy

### 4.1 Synergistic potential of combining targeted therapy with oncolytic viruses

Targeted therapy has emerged as a leading approach in anti-tumor treatment, offering the potential for more precise and effective interventions. The combination of targeted therapy with oncolytic viruses represents a compelling frontier for exploration and holds significant promise in the ongoing quest for improved cancer treatments. Targeted therapy, designed to specifically target cancer cells by interfering with specific molecules involved in tumor growth and progression, has revolutionized the landscape of cancer treatment. By honing in on the unique molecular features of cancer cells, targeted therapies aim to disrupt the signaling pathways critical for tumor survival and proliferation, while sparing normal cells from unnecessary damage. When combined with oncolytic viruses, which possess the ability to selectively replicate within tumor cells, targeted therapy can potentially synergize to enhance anti-tumor effects. The precision of targeted therapy aligns well with the selective nature of oncolytic viruses, creating an opportunity for a dual-pronged attack on cancer. Moreover, the use of targeted therapy may help sensitize cancer cells to the oncolytic effects of viruses, thereby augmenting their overall efficacy. For instance, Nguyen et al. (Nguyen et al., 2021) indicated that JAK inhibitors offer a dual advantage by not only suppressing the antiviral immune response to enhance the oncolytic effect of oncolytic viruses (OVs) but also mitigating the issue of T-cell exhaustion resulting from chronic inflammation. Also, Patel et al. (Patel et al., 2019) suggested that the combined administration of ruxolitinib and VSV-IFNβ therapy exhibited a promising trend towards enhanced mouse survival, while minimally impacting programmed death-ligand 1 (PDL-1) levels and immune infiltration within the tumor. These findings provide compelling support for the necessity of further clinical assessment of the combination approach involving JAK/STAT inhibition and virotherapy. In glioma cell lines, Du et al. (Du et al., 2012) showed that inhibition of the IKK/NF-kB signaling pathway using the NF-kB kinase inhibitor TPCA-1 has been demonstrated to effectively diminish type I interferon-mediated antiviral responses. Moreover, various targeted drugs, including MEK inhibitors (Bommareddy et al., 2018), the PI3K inhibitor BKM120 (Wang et al., 2019), and pertuzumab (Yoo et al., 2014), have exhibited the capacity to augment viral replication in the context of viral therapy. In addition, some targeted drugs have been shown to promote virus activation and the host’s anti-tumor immunity or to inhibit immune suppressor factors in the tumor microenvironment. Hutzen et al. (Hutzen et al., 2017) found that TGF-β inhibition,A8301, can augment the immunotherapeutic efficacy of oncolytic herpes virotherapy. And Yoo et al. (Yoo et al., 2016) showed that combination treatment also significantly enhanced NK cell activation and adjuvant NK cell therapy of mice treated with bortezomib and oHSV improved anti-tumor efficacy. Besides rituximab combined with oncolytic viruses, can enhance NK-mediated cytotoxicity and treat chronic lymphocytic leukemia (Parrish et al., 2015).

### 4.2 Enhanced antitumor effects through anti-angiogenic therapy

Anti-angiogenic therapy has been shown to promote tumor vascular normalization and remodel the tumor microenvironment from an immune-suppressive state. In their study, Malfitano et al. (Malfitano et al., 2020) reported that treatment with vascular endothelial growth factor (VEGF) can modulate the release of cytokines, including IL-1β, IL-6, and CXCL1, thereby disrupting the immune homeostasis of the tumor microenvironment and creating an environment conducive to immune-based therapies. Similarly, Saha et al. (Saha et al., 2018) observed that systemic TKI (axitinib) synergistically enhances the antitumor efficacy of G47Δ-mIL12 in both immunodeficient and immunocompetent orthotopic GBM models, resulting in increased macrophage infiltration, extensive tumor necrosis, and inhibition of the PDGFR/ERK pathway.

## 5 The combination of oncolytic virotherapy and cell therapy

### 5.1 Synergistic potential of oncolytic viruses and cell therapy in cancer treatment

The integration of oncolytic viruses and cell therapy represents a promising approach in cancer treatment (Mamola et al., 2023). Cell therapy, which harnesses immune cells to selectively eliminate cancer cells, exhibits significant potential in managing resistant cancer types. When combined with oncolytic viruses, the synergistic effects of these modalities have the potential to enhance anti-tumor responses. Oncolytic viruses possess the ability to induce tumor cell lysis and establish an immunogenic microenvironment within the tumor, thereby stimulating the immune system. Conversely, cell therapy involves engineering immune cells to specifically recognize and attack cancer cells. The combined approach aims to orchestrate a comprehensive assault against cancer cells, targeting both the tumor cells themselves and the mechanisms that enable immune evasion. By leveraging the immune-stimulating properties of oncolytic viruses, the immune system can be primed for a more robust response, while cell therapy provides a potent and selective means of targeting cancer cells. Furthermore, the combination of oncolytic viruses and cell therapy holds promise in overcoming individual limitations. For instance, oncolytic viruses may sensitize cancer cells to the effects of cell therapy, and cell therapy can counteract the immunosuppressive nature of the tumor microenvironment.

### 5.2 Advancements in combination immunotherapy with oncolytic viruses and cell therapy

The combination approach of oncolytic viruses and cell therapy, two pivotal branches of immunotherapy, has garnered significant attention. A recent study demonstrated that IL21-armed recombinant oncolytic vaccinia virus exhibited robust anti-tumor effects both as a monotherapy and in conjunction with other immunotherapies (Chen et al., 2021). While this combination strategy has shown promising therapeutic outcomes in melanoma, lymphoma, and hematological diseases, its application in malignant epithelial tumors faces challenges such as target identification, cell infiltration, and tumor heterogeneity (Shi et al., 2020). In another investigation (Park et al., 2020), researchers proposed an innovative combination immunotherapy utilizing oncolytic viruses to enhance *de novo* CAR T cell targeting of solid tumors. The study revealed that OV19t induced local immunity characterized by the infiltration of endogenous and adoptively transferred T cells into the tumor. Moreover, CAR T cell-mediated tumor killing resulted in the release of virus particles from dying tumor cells, leading to increased expression of CD19t within the tumor microenvironment. Additionally, the exploration of oncolytic viruses combined with autologous cell infusion has provided novel insights into combination therapy. Zheng et al. (Zheng et al., 2022) unexpectedly discovered a synergistic effect between T cells and myxoma virus, which promoted autosis of solid tumor cells and facilitated tumor clearance. Their findings showed that T cell-derived interferon γ (IFNγ)-protein kinase B (AKT) signaling synergized with myxoma virus-induced M-T5-SKP-1-VPS34 signaling, triggering robust autosis of tumor cells. In the realm of immunotherapy, natural killer (NK) cells possess inherent advantages over T cells. NK cells have multiple sources and their recognition ability is not contingent upon human leukocyte antigen matching, thereby reducing the risk of graft-versus-host disease. However, the application of NK cells or CAR-NK cells in combination with oncolytic virus therapy is still in the exploratory stage. Preclinical studies have demonstrated that the utilization of NK cells overexpressing CCR5 in combination with oncolytic virus therapy exhibited superior efficacy compared to monotherapy in a mouse colon cancer model, and this combination approach significantly enhanced the infiltration of NK cells into the tumor microenvironment in comparison to the wild-type virus (Li et al., 2020). Another preclinical study highlighted that the combination of an oncolytic virus expressing the IL15/IL15Rα complex with frozen, ready-to-use EGFR-CAR NK cells elicited potent antitumor responses in glioblastoma (Ma et al., 2021).

## 6 Prospects

Currently, the integration of oncolytic viruses with other therapeutic approaches is a critical focus in the advancement of oncolytic virus research. However, the optimal combination is not merely the sum of its parts; it necessitates thoughtful assessment of the synergistic functions of the drugs involved. Additionally, precise classification of combination strategies based on tumor type, stage, and the mechanism of each drug is imperative. For instance, certain early-stage tumors may respond effectively to oncolytic viruses alone, and the addition of other drugs might introduce unwanted side effects, potentially diminishing treatment efficacy. Conversely, late-stage tumors that are unresponsive to singular treatments may benefit from the combination of oncolytic viruses with other drugs, aiming to achieve superior therapeutic outcomes. Moreover, different tumor types and stages warrant tailored combination strategies. Malignant tumors, for example, may be suppressed by chemotherapy but at the cost of damaging healthy cells, resulting in adverse reactions. In such cases, combining oncolytic viruses with chemotherapy could leverage the specificity of oncolytic viruses to target tumor cells while minimizing the impact on normal cells from chemotherapy. Therefore, the accurate design of combination strategies not only enhances treatment effectiveness but also mitigates adverse effects, providing improved therapeutic options for cancer patients. In conclusion, the combined application of oncolytic viruses with traditional treatments is worthy of further exploration.
